# Glyoxalase I reduces glycative and oxidative stress and prevents age-related endothelial dysfunction through modulation of endothelial nitric oxide synthase phosphorylation

**DOI:** 10.1111/acel.12204

**Published:** 2014-02-24

**Authors:** Airi Jo-Watanabe, Takamoto Ohse, Hiroaki Nishimatsu, Masao Takahashi, Yoichiro Ikeda, Takehiko Wada, Jun-ichi Shirakawa, Ryoji Nagai, Toshio Miyata, Tetsuo Nagano, Yasunobu Hirata, Reiko Inagi, Masaomi Nangaku

**Affiliations:** 1 Division of Nephrology and EndocrinologyTokyo, Japan; 2 Department of Urology, the University of TokyoTokyo, Japan; 3 Department of Cardiovascular Medicine, the University of Tokyo, Graduate School of MedicineTokyo, Japan; 4 Laboratory of Food and Regulation Biology, Department of Bioscience, School of Agriculture, Tokai UniversityKumamoto, Japan; 5 Graduate School of Medicine, Tohoku UniversityMiyagi, Japan; 6 Graduate School of Pharmaceutical Sciences, the University of TokyoTokyo, Japan; 7 Department of Advanced Clinical Science and Therapeutics; 8 Chronic Kidney Disease Pathophysiology, The University of Tokyo Graduate School of MedicineTokyo, Japan

**Keywords:** advanced glycation end-products, aging, endothelial dysfunction, endothelial nitric oxide synthase, glycation, glyoxalase I

## Abstract

Endothelial dysfunction is a major contributor to cardiovascular disease (CVD), particularly in elderly people. Studies have demonstrated the role of glycation in endothelial dysfunction in nonphysiological models, but the physiological role of glycation in age-related endothelial dysfunction has been poorly addressed. Here, to investigate how vascular glycation affects age-related endothelial function, we employed rats systemically overexpressing *glyoxalase I* (*GLO1*), which detoxifies methylglyoxal (MG), a representative precursor of glycation. Four groups of rats were examined, namely young (13 weeks old), mid-age (53 weeks old) wild-type, and *GLO1* transgenic (WT/*GLO1* Tg) rats. Age-related acceleration in glycation was attenuated in *GLO1* Tg rats, together with lower aortic carboxymethyllysine (CML) and urinary 8-hydroxydeoxyguanosine (8-OHdG) levels. Age-related impairment of endothelium-dependent vasorelaxation was attenuated in *GLO1* Tg rats, whereas endothelium-independent vasorelaxation was not different between WT and *GLO1* Tg rats. Nitric oxide (NO) production was decreased in mid-age WT rats, but not in mid-age *GLO1* Tg rats. Age-related inactivation of endothelial NO synthase (eNOS) due to phosphorylation of eNOS on Thr495 and dephosphorylation on Ser1177 was ameliorated in *GLO1* Tg rats. *In vitro*, MG increased phosphorylation of eNOS (Thr495) in primary human aortic endothelial cells (HAECs), and overexpression of GLO1 decreased glycative stress and phosphorylation of eNOS (Thr495). Together, GLO1 reduced age-related endothelial glycative and oxidative stress, altered phohphorylation of eNOS, and attenuated endothelial dysfunction. As a molecular mechanism, GLO1 lessened inhibitory phosphorylation of eNOS (Thr495) by reducing glycative stress. Our study demonstrates that blunting glycative stress prevents the long-term impact of endothelial dysfunction on vascular aging.

## Introduction

Vascular aging is accompanied by endothelial dysfunction, which refers to functional alterations of the normal endothelial phenotype of arteries, represented by impaired endothelium-dependent relaxation (EDR; Lakatta & Levy, 2003; Brandes *et al*., 2005; Seals *et al*., 2011). Endothelial dysfunction is a major contributor to cardiovascular disease (CVD), the leading cause of death, particularly in elderly people (Lloyd-Jones *et al*., 2010; Seals *et al*., 2011).

Glycation is a nonenzymatic chemical reaction between an amino group of proteins or nucleotides and a reducing sugar which leads to the formation of irreversible end-products (advanced glycation end-products: AGEs). Methylglyoxal (MG) is a representative AGE precursor which is derived from glycolysis or glucose degradation and reacts with protein, nucleotides and basic phospholipids (Thornalley, 2008). The major modification of proteins by MG takes place on arginine residues and results in the formation of MG-derived hydroimidazolone MG-H1 and argpyrimidine. *N*^ε^-(1-carboxyethyl) Lysine (CEL) is formed through the modification of lysine residues by MG (Wautier & Schmidt, 2004; Thornalley, 2008).

Methylglyoxal is highly reactive and cytotoxic, and its detoxification mechanisms are well conserved across many species. The glyoxalase system consists of glyoxalase I (GLO1), glyoxalase II, and reduced glutathione (GSH). As the glyoxalase system plays a major role in cellular defense against glycative and oxidative stress (Thornalley, 1993), its rate-limiting enzyme, GLO1, is a key enzyme that diverts MG from glycation reactions (Thornalley, 2003).

The role of AGEs and their precursors in causing endothelial dysfunction has been demonstrated using nonphysiological experimental models, such as exogenous administration or pharmacological reduction of AGEs and AGE precursors (Hallam *et al*., 2010; Freidja *et al*., 2012; Sena *et al*., 2012). However, whether and how natural acceleration of glycative and carbonyl stress with aging actually affect endothelial function has not been fully elucidated. Given that MG forms physiologically out of the glycolysis pathway and because GLO1 activity is present in all tissues of prokaryotic and eukaryotic organisms (Thornalley, 1993), we hypothesized that accelerated intracellular glycative stress potentiates endothelial dysfunction with aging under normal physiological conditions. To test this hypothesis, we employed rats which systemically overexpress *GLO1* (Kumagai *et al*., 2009; Ikeda *et al*., 2011) and investigated whether and how intracellular glycation state alters endothelial function with age, that is, influences vascular aging.

## Results

### Physiological parameters and blood analyses

Physiological parameters and blood analyses of the rats are listed in Tables 1, 2, and Table S1 (young, 13 weeks old; mid-age, 53 weeks old). Because AGEs are present in daily food intake, body weight and amount of food intake were measured. We also measured blood pressure, glucose tolerance and lipid metabolism, which are known to modulate endothelial function. None of these parameters significantly differed between wild-type (WT) and *GLO1* transgenic (*GLO1* Tg) rats of the same age.

**Table 1:** Physiological parameters of young/mid-age and WT/*GLO1* Tg rats

	Young	Mid-age	Age-factor	Genotype-factor
Age (week)				
WT	13.4 ± 0.2 (23)	52.8 ± 1.0* (53)	*P* < 0.0001	ns
*GLO1* Tg	13.9 ± 0.2 (10)	53.5 ± 1.1* (48)		
Body weight (g)				
WT	406 ± 13 (23)	754 ± 14* (53)	*P* < 0.0001	ns
*GLO1* Tg	454 ± 19 (10)	732 ± 12* (48)		
Food intake (g day^−1^)				
WT	34 ± 0.4 (9)	36 ± 1.0 (7)	ns	ns
*GLO1* Tg	36 ± 0.4 (4)	34 ± 0.8 (6)		
Systolic blood pressure (mmHg)				
WT	125 ± 5 (4)	132 ± 5 (7)	ns	ns
*GLO1* Tg	130 ± 2 (4)	129 ± 2 (11)		
Diastolic blood pressure (mmHg)				
WT	94 ± 6 (4)	103 ± 4 (7)	ns	ns
*GLO1* Tg	101 ± 2 (4)	99 ± 2 (11)		

Values are mean ± 1 SEM. Numbers of animals are shown in parentheses. Two-way ANOVA with post hoc Bonferroni correction was performed. The number of multiple comparison was 2.

* *P* < 0.0001, vs. young rats in the same genotype group.

**Table 2:** Glucose tolerance of mid-age WT/*GLO1* Tg rats

	Mid-age WT	Mid-age *GLO1* Tg
Fasting blood glucose (mg dL^−1^)	74 ± 7 (4)	70 ± 4 (6)
Fasting insulin (μIU mL^−1^)	58.3 ± 14.1 (4)	55.1 ± 10.7 (6)
AUC in oral glucose tolerance test [(mg dL^−1^)·min]	14037 ± 529 (4)	13015 ± 302 (4)

Values are mean ± 1 SEM. Numbers of animals are shown in parentheses. *T*-test was performed and no significant differences were seen between mid-age WT and *GLO1* Tg rats.

### Glyoxalase I expression and activity of the thoracic aorta were significantly up-regulated in *GLO1* Tg rats

**Figure 1:**
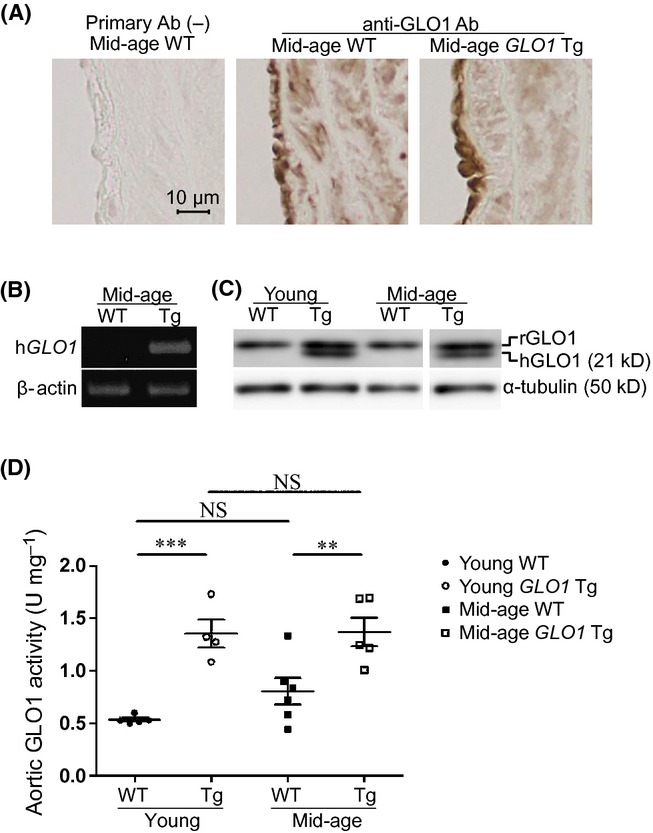
GLO1 expression and activity in thoracic aorta. Immunohistochemistry shows the same localization of GLO1 in thoracic aorta both in mid-age WT and *GLO1* Tg rats (A). Human *GLO1* was detected in thoracic aortas of *GLO1* Tg rats by polymerase chain reaction using specific primers for human *GLO1* (B) and by Western blotting using an antibody directed against both rat and human GLO1 (C). *GLO1* overexpression showed a 2.6- and 1.7-fold increase in GLO1 activity in thoracic aorta in young and mid-age rats, respectively (D). Young WT, *n* = 5; young *GLO1* Tg, *n* = 4; mid-age WT, *n* = 6; mid-age *GLO1* Tg, *n* = 5. Two-way ANOVA with post hoc Bonferroni correction was performed. The number of multiple comparison was 4. **P < 0.01, ***P < 0.001, vs. age-matched WT rats. NS indicates no significance.

We assessed GLO1 expression in thoracic aorta by immunohistochemistry. WT and *GLO1* Tg rats expressed GLO1 protein in cytoplasm of endothelial and smooth muscle cells (Fig. 1A), indicating that the localization of GLO1 in the groups was similar. Expression of human GLO1 in aortas of the *GLO1* Tg rats was confirmed at the transcription and protein levels (Fig. 1B,C). Overexpression of human *GLO1* resulted in an increase in GLO1 activity in the whole thoracic aorta of *GLO1* Tg rats compared with age-matched WT rats [0.53 ± 0.02 vs. 1.36 ± 0.14 U mg^−1^ (young WT vs. young *GLO1* Tg), *P* < 0.0001; 0.80 ± 0.13 vs. 1.37 ± 0.14 U mg^−1^ (mid-age WT vs. mid-age *GLO1* Tg), *P* < 0.0001; Fig. 1D].

### Age-related acceleration of glycative and oxidative stress was attenuated in aortic endothelium of *GLO1* Tg rats

No apparent histological changes were observed in the aortas of WT and *GLO1* Tg rats during the experimental period (Fig. S1). We then compared the effect of glycative stress on endothelial function in these rats in this absence of obvious structural changes.

**Figure 2:**
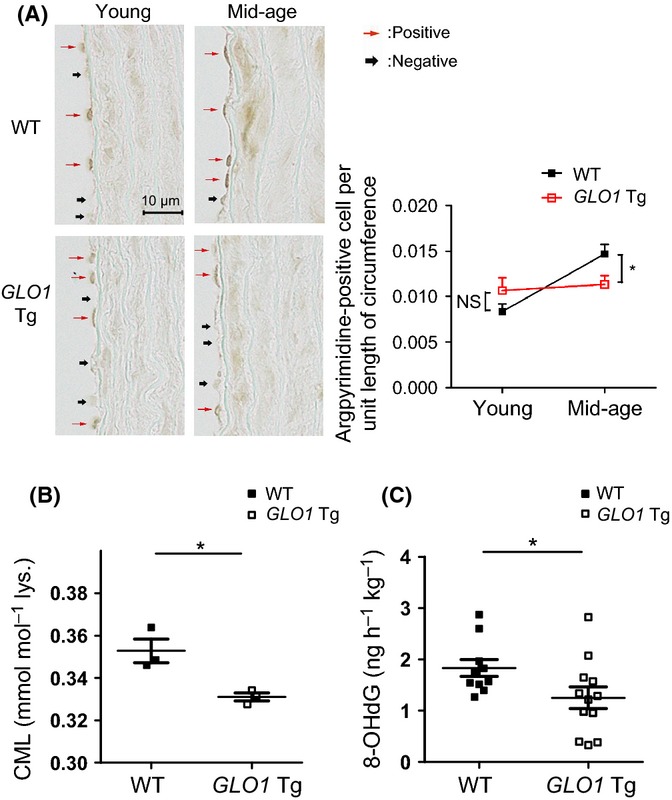
GLO1 attenuates methylglyoxal (MG) modification (A) and oxidative stress markers (B, C). (A) Immunohistochemical analysis of the MG-modified protein, argpyrimidine, showed the cytoplasmic and nuclear expression patterns of argpyrimidine in endothelium and media. The number of argpyrimidine-positive cells per unit length of circumference was used to express the level of MG-modified protein in endothelium. Age-related increase in MG-modified protein in endothelium was attenuated in *GLO-1* Tg rats. Young WT, *n* = 5; young *GLO1* Tg, *n* = 6; mid-age WT, *n* = 12; mid-age *GLO1* Tg, *n* = 9. Two-way ANOVA with post hoc Bonferroni correction was performed. The number of multiple comparison was 4. *P < 0.05, mid-age WT vs. *GLO-1* Tg rats. NS indicates no difference between young WT and *GLO1* Tg rats. (B) Glycative and oxidative stress marker, carboxymethyllysine, was decreased in mid-age *GLO1* Tg rats compared with mid-age WT rats. *n* = 3, each. *P < 0.05 by *t*-test. (C) 8-OHdG levels in urine over 24 h were measured by ELISA. Results indicates significantly lower levels of oxidative stress in mid-age *GLO1* Tg rats than WT rats. Mid-age WT, *n* = 10; mid-age *GLO1* Tg, *n* = 12. *P < 0.05 by *t*-test.

To evaluate the change in glycative stress status in thoracic aorta by *GLO1* overexpression, we measured the level of argpyrimidine, a marker of AGEs in these rats. Immunohistochemical analysis showed the cytoplasmic and nuclear expression patterns of argpyrimidine in endothelium and media. The number of argpyrimidine-positive endothelial cells per unit length of circumference significantly increased by aging in WT rats, and age-related MG modification was significantly attenuated in *GLO1* Tg rats (Fig. 2A).

We also assessed oxidative stress status because glycative stress and oxidative stress interact with each other, resulting in a vicious circle. Aortic carboxymethyllysine (CML), which is a glycative and oxidative stress marker, was significantly decreased in mid-age *GLO1* Tg rats compared with mid-age WT rats (Fig. 2B). Urinary 8-hydroxydeoxyguanosine (8-OHdG) levels were also reduced in mid-age *GLO1* Tg rats compared with mid-age WT rats (Fig. 2C). Other oxidation products, protein carbonyls and 4-hydroxy-2-nonenal (4-HNE), were significantly increased by mid-aged aortas in WT rats, but not in *GLO1* Tg rats (Fig. S2A–D). Together, these findings confirm that age-related acceleration of glycative and oxidative stress was decreased in *GLO1* Tg rats.

### Age-related endothelial dysfunction was ameliorated in *GLO1* Tg rats

**Figure 3:**
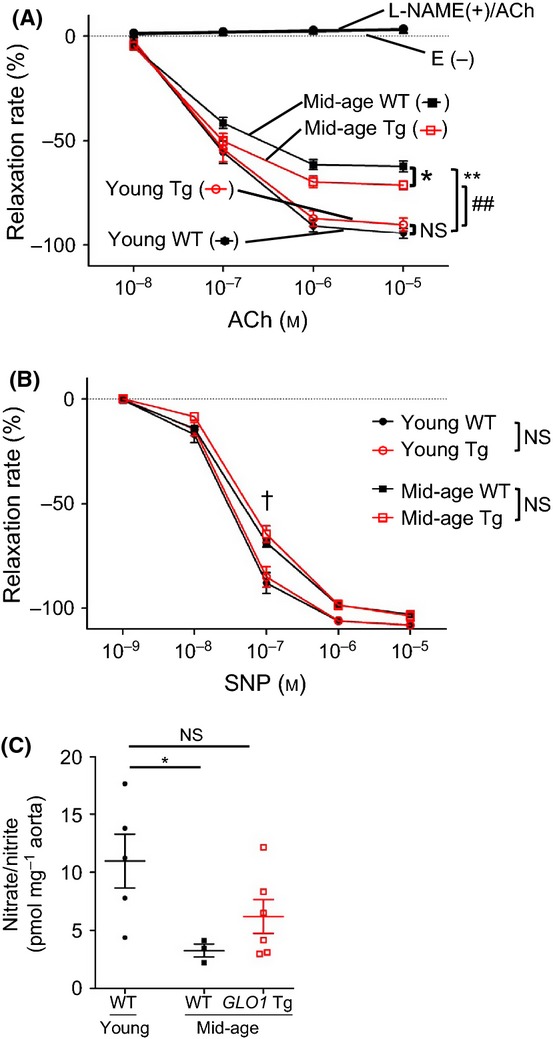
Dose-dependent relaxation curve for (A) acetylcholine (ACh) and (B) sodium nitroprusside (SNP), and (C) nitrate and nitrite production induced by *ex vivo* ACh stimulation. (A, B) Four aortic rings from each rat were used for experiments, two E+ rings and two E− rings. All four rings were treated with ACh, SNP, and *N*^G^-nitro-l-arginine methyl ester (L-NAME). Young WT, *n* = 5; young *GLO1* Tg, *n* = 6; mid-age WT, *n* = 12; and mid-age *GLO1* Tg, *n* = 9. (A) Endothelium-intact vessels (E+) of mid-age rats showed impaired ACh-induced vasorelaxation compared with those of young rats; **P < 0.0001, ^##^*P* < 0.0001 vs. young WT/*GLO1* Tg rats, respectively. Importantly, mid-age *GLO1* Tg rats showed significantly better relaxation responses than mid-age WT (−62.2 ± 2.6% vs. −71.2 ± 2.1%, *P < 0.05). The ACh dose responses of endothelium-deprived vessels (E−) and E+ vessels pretreated with L-NAME were completely abolished in all groups. Two-way ANOVA with post hoc Bonferroni correction was performed. The number of multiple comparison was 112. (B) Endothelium-independent vasorelaxation by SNP was attenuated by aging in both WT and *GLO1* Tg rats only at the concentration of 10^−7^ m, but the dose responses were not significantly different between WT and *GLO1* Tg rats. Two-way ANOVA with post hoc Bonferroni correction was performed. The number of multiple comparison was 30. ^†^*P* < 0.0001, young vs. mid-age rats in the same genotype group. (c) Nitrate/nitrite was used as an index of nitric oxide (NO) production by ACh stimulation. Aortic rings in Krebs–Henseleit buffer under continuous bubbling with 95% O_2_–5% CO_2_ were stimulated by 5 × 10^6^ m ACh. Nitrate/nitrite in Krebs–Henseleit buffer was measured before and after ACh stimulation. Nitrate/nitrite was expressed as pmol mg^−1^ wet weight of aortic rings. Nitrate/nitrite significantly diminished in mid-age WT rats, whereas mid-age *GLO1* Tg rats showed almost the same level of NO production as young WT rats. Young WT, *n* = 5; mid-age WT, *n* = 3; mid-age *GLO1* Tg, *n* = 6. *P < 0.05 by *t*-test, young vs. mid-age WT rats. NS indicates no significance.

We next focused on functional changes in the aorta associated with aging. For this purpose, we examined the endothelium-dependent and endothelium-independent vasorelaxation induced by acetylcholine (ACh) and nitroprusside (SNP), respectively. The endothelium-dependent relaxing agent ACh induced dose-dependent relaxation in endothelium-intact (E+) aortic rings of all groups (Fig. 3A). Mid-age E+ aortic rings demonstrated a significant decrease in response, as characterized by a lower maximal relaxation, compared with young E+ rings (−94.4 ± 2.5 in young WT vs. −62.2 ± 2.6% in mid-age WT, *P* < 0.0001). Whereas we could not detect a difference in ACh dose responses between young WT and *GLO1* Tg rats, mid-age *GLO1* Tg rats showed significantly better responses than mid-age WT rats (−62.2 ± 2.6 in mid-age WT vs. −71.2 ± 2.1% in mid-age *GLO1* Tg, *P* < 0.05). The responses to ACh of endothelium-removed (E−) vessels and E+ vessels pretreated with *N*^G^-nitro-L-arginine methyl ester (L-NAME) were completely abolished in all groups, indicating that the responses to ACh were endothelium- and NOS-dependent. Endothelium-independent vasorelaxation by a nitric oxide (NO) donor, SNP, was attenuated by aging in both WT and *GLO1* Tg rats only at the SNP concentration of 10^−7^ M, but the dose responses were not significantly different between age-matched WT and *GLO1* Tg rats (Fig. 3B). These results suggest that increased aortic GLO1 activity ameliorates age-related endothelial dysfunction in a NOS-dependent manner without changing vascular smooth muscle reactivity to NO.

### Age-related decrease in NO bioavailability was attenuated in *GLO1* Tg rats

As age-related reduction in ACh-induced vasorelaxation was ameliorated in *GLO1* Tg rats without a change in vascular smooth muscle reactivity to NO, we speculated that *GLO1* overexpression mitigated age-related endothelial dysfunction through increased net NO production by endothelium, or in other words NO bioavailability. We then measured nitrate and nitrite, which were converted from NO produced by ACh-stimulated aortas, to verify whether endothelium-derived NO bioavailability increased in mid-age *GLO1* Tg compared with mid-age WT rats. While nitrate and nitrite production by ACh stimulation was significantly diminished in mid-age WT rats, no significant decrease was detected in nitrate and nitrite production by mid-age *GLO1* Tg rats compared with that by young WT rats (Fig. 3C).

### NO quenching by superoxide did not differ between mid-age WT and *GLO1* Tg rats

We next explored the mechanism of this improvement in NO bioavailability in *GLO1* Tg rats. As NO bioavailability is dependent on the balance between its production by endothelial nitric oxide synthase (eNOS) and loss through reaction with superoxide, we next investigated the involvement of these factors.

To investigate whether increased NO bioavailability in mid-age *GLO1* Tg rats is attributed to decreased NO quenching by superoxide, we examined 3-nitrotyrosine formation in aortic tissue as a surrogate index of the reaction of NO with superoxide by immunohistochemistry and Western blotting. Immunohistochemical analysis showed that the 3-nitrotyrosine-positive area per unit length of the circumference of aorta in endothelium was almost identical between aged WT and *GLO1* Tg rats (Fig. S3A). Western blot analysis also showed no significant difference in protein nitrosylation in aorta between mid-age WT and *GLO1* Tg rats (Fig. S3B). These data suggest that improved NO bioavailability in mid-age *GLO1* Tg rats is attributable to increased production of NO by eNOS rather than excessive superoxide quenching of NO.

### Age-related change in phosphorylation of eNOS was attenuated in *GLO1* Tg rats

**Figure 4:**
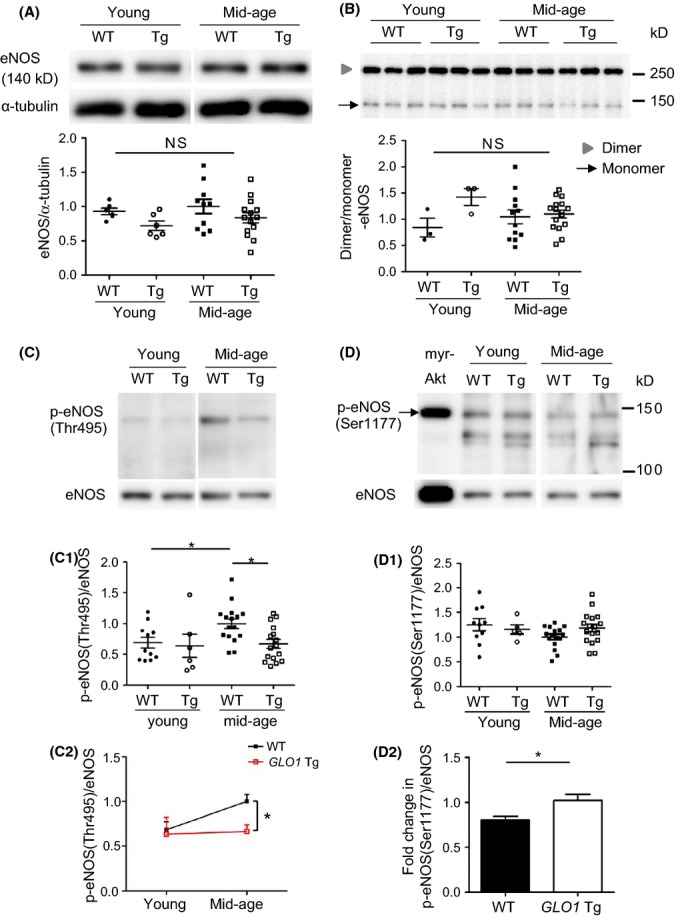
Western blotting of endothelial nitric oxide synthase (eNOS) and its phosphorylation. No significant difference was seen between four groups in eNOS/α-tubulin ratio (A: Young WT, *n* = 6; young *GLO1* Tg, *n* = 6; mid-age WT, *n* = 10; mid-age *GLO1* Tg, *n* = 14) or dimer/monomer ratio of eNOS (B: Young WT, *n* = 3; young *GLO1* Tg, *n* = 3; mid-age WT, *n* = 12; mid-age *GLO1* Tg, *n* = 16). NS indicates no significant difference by one-way ANOVA. (C) eNOS phosphorylation on Thr495 was elevated by aging in WT rats and was significantly reduced in *GLO1* Tg rats compared with mid-age WT rats (C-1). Age-related increase in p-eNOS (Thr495) was significantly attenuated in *GLO1* Tg rats (C-2). Young WT, *n* = 11; young *GLO1* Tg, *n* = 6; mid-age WT, *n* = 16; mid-age *GLO1* Tg, *n* = 16. Two-way ANOVA with post hoc Bonferroni correction was performed. The number of multiple comparison was 4. Phosphorylation on Ser1177 tends to decrease by aging in WT rats (D-1). Age-related dephosphorylation of eNOS (Ser1177) was significantly attenuated in *GLO1* Tg rats. *P < 0.05 by *t*-test (D-2). Young WT, *n* = 10; young *GLO1* Tg, *n* = 5; mid-age WT, *n* = 16; mid-age *GLO1* Tg, *n* = 17. Myr-Akt serves as a positive control for phosphorylation of eNOS on Ser1177.

To further investigate the mechanism of the improvement in NO bioavailability by GLO1, we assessed the change in eNOS expression and its activity by *GLO1* overexpression. No significant difference was seen between four groups in eNOS expression (Fig. 4A), nor in eNOS dimerization, which is indispensable for the expression of its enzymatic activity (Fig. 4B). We therefore focused on post-translational modification of eNOS, specifically phosphorylation, which is another critical factor for eNOS activity (Govers & Rabelink, 2001).

We examined eNOS phosphorylation on its activation (Ser1177) and inhibitory (Thr495) sites by Western blotting. Phosphorylation of eNOS on Thr495, which results in a decrease in eNOS activity, increased by aging in WT rats and mid-age *GLO1* Tg rats showed significantly reduced phosphorylation compared with mid-age WT rats (Fig. 4C-1). Whereas phosphorylation of eNOS on Thr495 did not significantly differ between young WT and *GLO1* Tg rats, age-related increase in phosphorylation on eNOS (Thr495) was significantly attenuated by *GLO1* overexpression (Fig. 4C-2). On the other hand, age-related decrease in phosphorylation on Ser1177 was ameliorated in *GLO1* Tg rats (Fig. 4D).

To elucidate the mechanism by which age-related phosphorylation/dephosphorylation of eNOS was altered in *GLO1* Tg rats, we examined the protein kinase C (PKC) family, which are upstream kinases leading to eNOS (Thr495) phosphorylation. Among many PKC isozymes, we focused mainly on PKCα and βII, which were reported to phosphorylate eNOS (Thr495) *in vitro* and *in vivo* (Chiasson *et al*., 2011; Xie *et al*., 2012). The expression of total PKCα did not significantly differ between mid-age WT and *GLO1* Tg rats (Fig. S4A). Further, the degree of phosphorylation of PKCα, PKC βII and pan PKC was also similar (Fig. S4B–D).

### GLO1 reduced glycative stress in association with the change in phosphorylation of eNOS in human aortic endothelial cells

To further elucidate the mechanism by which GLO1 ameliorates endothelial dysfunction, human aortic endothelial cells (HAECs) overexpressing GLO1 protein were utilized to assess whether and how GLO1 altered glycation status and phosphorylation of eNOS in *in vitro* studies.

**Figure 5:**
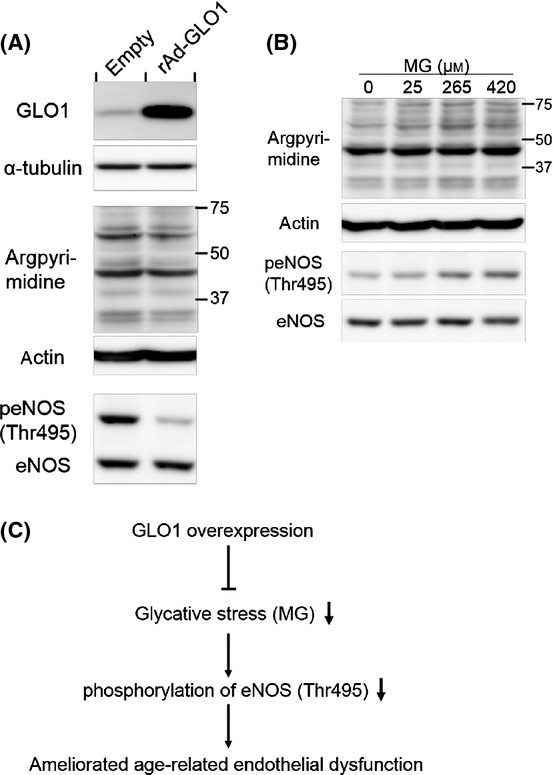
GLO1 ameliorated phosphorylation of endothelial nitric oxide synthase (eNOS) (Thr495) induced by methylglyoxal (MG) in association with lowering of glycative stress in human aortic endothelial cells (HAECs). (A) GLO1 overexpression decreased argpyrimidine and phosphorylation of eNOS (Thr495). rAd-GLO1 and empty indicate HAECs infected with the recombinant adenovirus vector with or without GLO1, respectively. HAECs were infected with at a multiplicity of infection (MOI) of 20. (B) On the other hand, MG increased phosphorylation of eNOS (thr495) in a dose-dependent manner in association with glycative stress. After starvation for 24 h, HAECs were treated with MG at final concentrations of 0, 25, 265, and 420 μm, for 24 h. (C) A schema describes the mechanism of GLO1 attenuates age-related endothelial dysfunction.

Overexpression of GLO1 in HAECs reduced basal level of glycation status and decreased inhibitory phosphorylation of eNOS on Thr495 (Fig. 5A). These *in vitro* data were supported by the results showing that eNOS phosphorylation state was altered in *GLO1* Tg rats.

### Glycative stress altered eNOS phosphorylation

Because overexpression of GLO1 reduced glycative stress and decreased phosphorylation of eNOS (Thr495), we hypothesized that GLO1 reduced inhibitory phosphorylation of eNOS (Thr495), which was induced by glycative stress. To corroborate these hypotheses, HAECs were then treated with various concentrations of MG to investigate whether glycative stress causes phosphorylation of eNOS on Thr495. Methylglyoxal indeed induced phosphorylation of eNOS (Thr495) as well as augmented glycative stress in the similar concentration-dependent manner (Fig. 5B). These results indicate that the mechanism, by which GLO1 overexpression reduced inhibitory phosphorylation of eNOS on Thr495, is attributable to detoxification of MG by GLO1 (Fig. 5C).

These data supported our *in vivo* data showing that GLO1 activates eNOS by decreasing age-related eNOS (Thr495) phosphorylation and eNOS (Ser1177) dephosphorylation, resulting in increased NO bioavailability and amelioration of endothelial dysfunction.

Taken together, we have demonstrated that, in *GLO1* Tg rats, age-related acceleration in glycative and oxidative stress was mitigated and age-related endothelial dysfunction was ameliorated, concomitant with attenuation of age-related eNOS (Thr495) phosphorylation and eNOS (Ser1177) dephosphorylation, resulting in increased NO bioavailability and amelioration of endothelial dysfunction. As a molecular mechanism, we have shown that GLO1 reduced inhibitory phosphorylation of eNOS (Thr495) caused by MG.

## Discussion

In this study, we have shown that aging is accompanied by the acceleration of glycative and oxidative stress in the vasculature and impairment of endothelium-dependent vasorelaxation and that the latter results from a decrease in eNOS activity due to phosphorylation of eNOS on Thr495 and dephosphorylation on Ser1177. Importantly, we further demonstrated using *GLO1* systemic overexpression model rats that regulation of glycative stress prevents these age-associated changes, namely vascular glycative and oxidative stress, glycation and carbonylation of proteins, eNOS phosphorylation, and endothelial dysfunction. These findings suggest that regulation of glycative stress is a promising strategy for the prevention of vascular aging.

Our findings in *in vivo* studies presented three mechanisms that were only tentatively linked: GLO1/glycation and functional changes in the aging aorta, GLO1 and eNOS phosphorylation pattern, and glycation and altered eNOS phosphorylation. Although our finding showed that *GLO1* overexpression did not change the upstream PKC signaling pathways leading to the phosphorylation of eNOS (Thr495), other protein kinases might play a role in altered eNOS phosphorylation in *GLO1* Tg rats. Another possibility is that GLO1 mitigates the inactivation of eNOS via post-translational modification, such as aging-induced glycation of eNOS itself. To elucidate the mechanism for the findings in *in vivo* studies, we performed cellular studies and successfully demonstrated the cause and effect between GLO1 and eNOS phosphorylation pattern, as well as glycation and altered eNOS phosphorylation. GLO1 overexpression in HAECs reduced inhibitory phosphorylation of eNOS (Thr495), and MG induced phosphorylation of eNOS (Thr495). Although we have not succeeded in detecting increase in eNOS activity by GLO1 overexpression, studies have reported that phosphorylation of eNOS (Thr495) negatively regulates eNOS activity. Therefore, it is highly likely that GLO1 activates eNOS by attenuating age-related increase in inhibitory phosphorylation of eNOS (Thr495) and ameliorates age-related endothelial dysfunction.

It has been demonstrated that endothelial dysfunction, as indexed by impaired flow-mediated vasodilatation, becomes evident in apparently healthy middle-aged men (Lakatta & Levy, 2003). To focus on early arterial functional changes that become evident much sooner than the other phenotypic or morphological changes associated with CVD, such as hypertension and atherosclerosis (Lakatta & Levy, 2003), we used 53-week-old middle-aged rats in the present study. In this setting, *GLO1* overexpression ameliorated age-related endothelial dysfunction without changing conventional CVD risk factors such as body weight, blood pressure, glucose tolerance, or lipid metabolism, firmly indicating that GLO1 reduces glycative and oxidative stress and essentially ameliorates age-related endothelial dysfunction.

The glyoxalase system and GLO1 are highly conserved through a wide range of species and various kinds of cells and tissues as defensive system against harmful carbonyl stress, and many studies have demonstrated the role of AGEs and glycative stress in vascular function. Unfortunately, however, most of those studies utilized nonphysiological animal models that do not mimic human physiological aging and are therefore unable to answer the question of how glycative stress affects vascular function physiologically. In contrast, we utilized a model of moderate overexpression of GLO1 activity and investigated the natural course of vascular aging without additional induction of disease. We have also reported the protective effects of GLO1 against kidney aging (Ikeda *et al*., 2011). Our studies are more closely directed to the physiological role of glycative stress and importance of its regulation.

In summary, we demonstrated that GLO1 reduces age-related glycative and oxidative stress in the vasculature and attenuates endothelial dysfunction, concomitant with eNOS activation due to reduced age-related phosphorylation/dephosphorylation of eNOS from early aging. As a molecular mechanism, we have shown that GLO1 reduced inhibitory phosphorylation of eNOS (Thr495) caused by MG. Importantly, we provided evidence for that the regulation of glycative stress by enhanced GLO1 activity counteracts physiological vascular aging. Given findings that GLO1 mitigates aging-associated endothelial dysfunction, which causes CVD in the long term, regulation of glycative stress may represent a versatile target for the prevention of vascular aging and CVD.

## Experimental procedures

### *GLO1* transgenic rats and genotyping

*GLO1* transgenic (*GLO1* Tg) rats were generated as described previously (Inagi *et al*., 2002). Briefly, to generate the human *GLO1* transgenic construct, the entire coding sequence of human *GLO1* cDNA was cloned into the EcoR I site of the pBsCAG-2. The *GLO1* transgene isolated by digestion of pBsCAG-2 containing *GLO1* cDNA with Kpn I and Sac I was microinjected into one pronucleus of fertilized Wistar rat oocytes, which were then transferred into the oviducts of pseudopregnant rats.

The genotypes of rats were determined by polymerase chain reaction (PCR) of genomic DNA extracted from tail tissue using an REDExtract-N-Amp Tissue PCR kit (R4775; Sigma, St. Louis, MO, USA). As specific primers for *GLO1* vector, primers for cytomegalovirus enhancer, sense (5′-GTC GAC ATT GAT TAT TGA CTA G-3′) and antisense (5′-CCA TAA GGT CAT GTA CTG-3′), were used to amplify a 320-bp fragment. Polymerase chain reaction amplification was carried out with initial denaturation at 94 °C for 3 min, followed by 35 cycles of 94 °C for 30 s, 55 °C for 30 s and 72 °C for 30 s.

### Design of *in vivo* experiments

All procedures were carried out in accordance with institutional guidelines for animal experimentation. All experiments involving rats were reviewed and approved by the Ethics Committee for Animal Care and Use of the University of Tokyo, Tokyo, Japan (P11-009).

Male *GLO1* Tg rats and wild-type (WT) littermates were studied at 13 and 53 weeks of age. Animals were housed in the same environment, allowed free access to food and water, and exposed to a 12-h light/dark cycle. Body weight, blood pressure, glucose tolerance, and plasma lipid metabolism were measured. Thoracic aorta was analyzed for expression and activity of GLO1, MG-modified protein level, CML level, protein carbonyls, vascular function, NO production, and protein levels of total and phosphorylated eNOS. Urine samples were collected for measurement of 8-OHdG level.

### Measurement of blood pressure and blood parameters

For blood pressure measurements, rats were kept in a quiet room at 25 °C. Blood pressure was measured by the tail-cuff method using a blood pressure analyzer (BP-98A; Softron, Tokyo, Japan). Blood glucose was measured with an automated blood glucose meter (Glutest Ace; Sanwa Chemical, Nagoya, Japan). Plasma insulin level was measured using a rat insulin ELISA kit (AKRIN-010T; Shibayagi, Gunma, Japan) according to the manufacturer’s instructions. Serum triglyceride, total cholesterol, LDL cholesterol, and HDL cholesterol were measured at Bio Medical Laboratories, Inc (Tokyo, Japan).

### Oral glucose tolerance test

The oral glucose tolerance test was performed 5 days before euthanization. The rats were fasted overnight (12 h) before the test. After baseline glucose level was measured and plasma was collected for insulin measurement, glucose (2.0 g kg^−1^ body weight) was administered to the rats as a 20% solution with a feeding cannula. Blood samples were collected at 0, 15, 30, and 120 min after glucose administration.

### Aorta preparation

The rats were heparinized 10–30 min before the rats were killed. After rats were anesthetized with 50 mg kg^−1^ IP pentobarbital and euthanized by bleeding, the thoracic aorta, from the distal aortic arch to the descending thoracic aorta at the level of the diaphragm, was dissected and immediately placed in a culture dish containing Krebs–Henseleit buffer (K3753; Sigma-Aldrich) gassed with 95% O_2_–5% CO_2_ at 37 °C. The aorta was then cleaned of surrounding connective tissue and sectioned into rings for further experiments.

Aortic sections were fixed in 10% neutral-buffered formalin or embedded in OCT compound (4583; Tissue-Tek, Tokyo, Japan) for immunohistochemical analysis, snap-frozen in liquid nitrogen for polymerase chain reaction (PCR), GLO1 activity assay, or immunoblotting, or kept in Krebs–Henseleit buffer at 37 °C under continuous bubbling with 95% O_2_–5% CO_2_ for isometric wall tension studies and measurement of NO production.

### Reverse transcription and PCR

Total RNA was extracted from aortic tissues by the acid guanidinium thiocyanate-phenol-chloroform extraction method (RNAiso Plus, 9109; Takara Bio, Shiga, Japan). RNA concentrations were determined using a BioSpec-nano spectrophotometer (Shimadzu, Kyoto, Japan). Reverse transcription was performed with 500 ng of total RNA using the PrimeScript(R) RT Master Mix (RR036B; Takara) according to the manufacturer’s instructions. The primers used for PCR were as follows: human *GLO1* 5′-CAGGCTAGCCATGGCAGAACCGCAGCC-3′ (forward), 5′-GGAGAATTCTCACAGCACTACATTAAG-3′ (reverse); rat *actin beta* 5′-CTTTCTACAATGAGCTGCGTG-3′ (forward), 5′-TCATGAGGTAGTCTGTCAGG-3′ (reverse).

Polymerase chain reaction was performed on 1 μL per well of reverse-transcribed product (50 ng total RNA) using GoTaq Green Master Mix (M7123; Promega, Madison, WI, USA). PCR amplification was carried out with initial denaturation at 95 °C for 30 s, followed by 35 cycles of 95 °C for 30 s, 62 °C for 30 s, and 72 °C for 30 s.

### Measurement of GLO1 activity

Thoracic aorta (50 mg) was chopped and lysed with 250 μL lysis buffer (10 mm sodium phosphate buffer with 0.02% Triton X-100, pH 7.0), sonicated for 2 min and centrifuged at 20 000 *g* for 20 min at 4 °C. The supernatant was used for measurement of GLO1 activity as described previously (Kumagai *et al*., 2009). Briefly, 2 μL of the supernatant was added to the mixture containing 5 μL of 40 mm MG, 5 μL of 40 mm GSH and 90 μL of 50 mm sodium phosphate buffer, pH 6.6, which was preincubated at 37 °C. Increase in absorbance at 240 nm due to the formation of S-D-lactoylglutathione was monitored for 90 s and corrected with protein concentration. As a measurement standard, recombinant human glyoxalase I (4959-GL-02M, R&D Systems, Minneapolis, MN, USA) was used. GLO1 activity was expressed as U per mg protein.

### Measurement of carboxymethyllysine in aorta

Protein was precipitated with 20% trichloroacetic acid. Standard [^2^H_2_]CML (PolyPeptide Laboratories, Strasbourg, France) and [^13^C_6_]Lysine (Cambridge Isotope Laboratories, Inc., Tewksbury, MA, USA) were added to the pellets, which were hydrolyzed with 1 mL of 6 N HCl at 100 °C for 18 h. The dried sample was resuspended in H_2_O and passed over a Strata-X-C column (Phenomenex, Torrance, CA, USA) which had been prewashed with 1 mL methanol and equilibrated with 1 mL H_2_O. Then, the column was washed with 3 mL of 2% formic acid and eluted with 3 mL of 7% ammonia. The pooled elution fractions were dried and resuspended in 1 mL of 20% acetonitrile containing 0.1% formic acid for liquid chromatography–tandem mass spectrometry (LC-MS/MS) analysis.

Samples were assayed by LC-MS/MS using a TSQ Vantage triple stage quadrupole mass spectrometer (Thermo Scientific, Waltham, MA, USA). LC was conducted on a ZIC®-HILIC Column (150 × 2.1 mm, 5 μm). The mobile phase was solvent A, H_2_O containing 0.1% formic acid, and solvent B, acetonitrile containing 0.1% formic acid. The flow rate was 0.2 mL min^−1^ and the column was kept at 40 °C. Retention time for CML and lysine was approximately 12 and 13 min, respectively. CML, lysine, and standard were detected by electrospray positive ionization–mass spectrometric multiple reaction monitoring. The ionization source temperature was 200 °C and the ion capillary temperature 300 °C. A collision-induced dissociation was performed using argon as the collision gas at a pressure of 1.2 mTorr. Programmed molecular ion and fragment ion masses and collision energies were optimized for multiple-reaction-monitoring detection of analytes.

### Measurement of 24-h urinary 8-OHdG level

Twenty-four-hour urine samples were collected for mid-age WT and *GLO1* Tg rats. All samples were centrifuged at 2000 g for 10 min to eliminate debris and then stored at −80 °C until further analysis. After centrifugation at 14 000 *g* for 30 min through molecular weight cutoff filters (Amicon Ultra 0.5 mL centrifugal filters, MWCO 10 kDa, UFC501096; Millipore Corp, Billerica, MA, USA), urinary 8-OHdG level was measured using a commercially available ELISA kit (Highly Sensitive ELISA Kit for 8-OHdG, Japan Institute for the Control of Aging; Nikken Seil Co., Ltd, Shizuoka, Japan). Urinary 8-OHdG level was expressed in ng h^−1^ kg^−1^ body weight.

### Immunohistochemistry

The following primary antibodies were used: polyclonal rabbit anti-GLO1 IgG [12 μg mL^−1^, obtained from immunization against synthetic peptide of rat GLO1, GIAVPDVYEA, which cross-reacts with the human GLO1 epitope (Ikeda *et al*., 2011)]; monoclonal mouse anti-argpyrimidine IgG (N213430, 1:100) from the Japan Institute for the Control of Aging; and polyclonal rabbit anti-3-nitrotyrosine IgG (N0409, 1:300) from Sigma-Aldrich. As a secondary antibody, biotinylated horse anti-mouse IgG antibody (BA-2001, 1:500) or goat anti-rabbit IgG antibody (BA-1000, 1:500) from Vector Laboratories (Burlingame, CA, USA) was used as appropriate.

Formalin-fixed, paraffin-embedded tissues were cut into 3-μm-thick sections and deparaffinized with Histoclear (National Diagnostic, Atlanta, GA, USA), rehydrated in graded alcohol and processed for antigen retrieval using 10 mm citric acid buffer (pH 6.0) or 1 mm EDTA (pH 8.0) in a microwave, followed by endogenous peroxidase blocking with 3% hydrogen peroxide, general protein blocking with blocking solution (2% normal horse serum, 1% BSA, 0.1% fish gelatin, and 0.1% sodium azide), and avidin/biotin blocking. The sections were incubated overnight at 4 °C with primary antibodies diluted in PBS containing 1% BSA. The sections were subsequently incubated with the secondary antibody, followed by incubation with Vectastain ABC reagent (PK-7100; Vector Laboratories). Staining color was developed by diaminobenzidine. Omission of the primary antibody served as control.

For quantification of immunohistochemistry, sections stained in each single experiment were used for analysis. The entire circumference of the aorta was studied for each rat. Whole analysis was performed with genomic information concealed. Images were processed and quantified with imagej software (National Institute of Health, Bethesda, MD, USA). For argpyrimidine immunohistochemistry, each endothelial cell was determined as positive or negative, and the number of positive endothelial cells was divided by the circumference. Although MG-H1 is a major MG adduct, argpyrimidine has been more studied and is a useful marker of MG-induced glycative stress. In this study, we utilized well-characterized antibody against argpyrimidine.

### Western blotting

Whole thoracic aorta was snap-frozen in liquid nitrogen and lysed with 300 μL of lysis buffer containing 50 mm Tris, pH 8.0, 5 mm EDTA, 150 mm NaCl, 1% Triton X-100, 1% Nonidet P-40, with protease and phosphatase inhibitors (Complete Mini #11836153001 and PhosSTOP #04906845001; Roche Applied Science, Mannheim, Germany). After sonication for 1 min and centrifugation at 16 000 *g* for 15 min, the supernatants were collected and the protein concentration was determined with a DC protein assay kit (500-0116; Bio-Rad, Richmond, CA, USA). Crude protein samples were separated by sodium dodecyl sulfate-polyacrylamide gel electrophoresis (SDS-PAGE) under reducing conditions and transferred to polyvinylidene difluoride (PVDF) membranes. The membranes were probed at 4 °C overnight with polyclonal rabbit anti-GLO1 antibody at 1:1000 dilution; polyclonal rabbit anti-phospho-eNOS (Thr-495 or Ser-1177) antibodies at 1:500 dilution (#9574 and #9571, respectively; Cell Signaling Technology, Beverly, MA, USA); polyclonal rabbit anti-PKCα at 1:1000 dilution; phospho-PKC α/βII (Thr638/641) at 1:1000 dilution; phospho-PKC (pan) (βII Ser660) antibodies at 1:1000 dilution (#2056, #9375, and #9371; Cell Signaling Technology); monoclonal mouse anti-4-HNE antibody (MHN-020P, Japan Institute for the Control of Aging) at 1:30 dilution; or anti-α-tubulin antibody (T6199; Sigma-Aldrich). Membranes probed with anti-phospho-eNOS antibody were then stripped and reprobed for monoclonal mouse anti-eNOS/NOS type III antibody at 1:1000 dilution (N30020; BD Transduction Laboratories, Lexington, KY, USA). Results were quantified by densitometry, and the ratio of phospho to total eNOS was determined in arbitrary units and expressed relative to the ratio for mid-age WT rats prepared and blotted at the same time.

As multiple bands were detected in Western blotting of phospho-eNOS (Ser1177), Human Umbilical Vein Endothelial Cells were infected with adenovirus overexpressing constitutively activated Akt1 (myr-Akt) and the cell lysate was used as a positive control.

For the detection of protein carbonyls as an oxidative marker, commercially available protein carbonyls Western blot detection kit (#ROIK03; SHIMA Laboratories Co., Ltd, Tokyo, Japan) was used. Membranes were incubated with 2,4-dinitrophenylhydrazine (DNPH) solution, to form the stable derivative of carbonyl groups. The derivatives were detected by anti-DNP antibody.

To distinguish the monomeric from the dimeric form of eNOS, the latter of which forms a stable SDS-resistant homodimer under reduced conditions at low temperature, low temperature SDS-PAGE (LT-PAGE)/Western blot was performed according to a published method (Klatt *et al*., 1995; Kakoki *et al*., 2000) with minor modification. Briefly, Laemmli buffer was added to protein extracts, and the mixture was stored at 4 °C to preserve the dimeric form under the reduced condition. Gels and buffers were equilibrated at 4 °C before electrophoresis, and the buffer tank was placed in an ice bath during the electrophoresis to keep the temperature of the gel below 15 °C. The proteins were transferred to PVDF membrane, and the membrane was probed with anti-eNOS antibody. The ratio of eNOS dimers to monomers was quantified by densitometry.

### Vascular isometric wall tension studies

Vascular responses of the thoracic aorta from four groups, young WT (*n* = 5), young Tg (*n* = 6), mid-age WT (*n* = 12) and mid-age Tg (*n* = 9), were tested in organ chambers. The thoracic aorta was excised and dissected free from connective tissue under a stereoscopic microscope. The endothelium was kept intact (E+) or was physically removed (E−) by gentle rubbing with a twist of cotton. Aortic rings (5 mm in length) were mounted horizontally between two stirrups in organ chambers filled with 20 mL modified Krebs–Henseleit buffer at 37 °C, under continuous bubbling with 95% O_2_–5% CO_2_. One stirrup was connected to an anchor and the other to a force transducer (T7-30-240; Orientec Co., Tokyo, Japan) to record isometric tension. The aortic rings were incrementally stretched to 1 gf (9.8 g·m s^−2^) for basal tension and precontracted with 10^−6^ m-norepinephrine, and cumulative dose responses to ACh (acetylcholine chloride, A6625; Sigma-Aldrich) and sodium nitroprusside (SNP) (sodium nitroprusside dihydrate, 71778; Sigma-Aldrich) were studied in the presence or absence of the vascular endothelium. To confirm the involvement of NOS in ACh-induced vasodilation, responses to ACh in the presence of an NOS inhibitor, 10^−3^ m *N*^G^-nitro-l-arginine methyl ester (L-NAME) (N412; Dojindo Molecular Technologies, Inc., Kumamoto, Japan), were also studied. For each animal, the dose–response curve was derived from the means of two aortic rings. The aortic responses were analyzed by two-way ANOVA with post hoc Bonferroni correction. Vasorelaxation was expressed as relaxation rate, as calculated by the percent decrease in tension from the norepinephrine-induced precontraction.

### Measurement of NO production by acetylcholine-stimulated aorta

Production of nitrate and nitrite is often used as markers of NO production. About 30 mg (wet weight) of endothelium-intact aortic segments was equilibrated in 2-mL plastic tubes that contained 1 mL of Krebs–Henseleit buffer for 30 min at 37 °C, under continuous bubbling with 95% O_2_–5% CO_2_. At the end of the incubation period, ACh (final concentration: 5 × 10^−6^ m) was added to the buffer, and the segments were allowed to stand for 5 min. The supernatant Krebs–Henseleit buffer was collected just before and after incubation with ACh to estimate NO production by ACh-stimulated aortic endothelial cells. Total nitrite (nitrate/nitrite) produced by ACh-stimulated aorta was measured with a nitrate/nitrite fluorometric assay kit (780051; Cayman Chemical Company, Ann Arbor, MI, USA).

### Cell culture

Primary HAECs (CC-2535; Lonza, Walkersville, MD, USA) were cultured in Endothelial Cell Growth Medium-2 (EGM-2) (CC-3162; Lonza). Subconfluent HAEC monolayers were passaged with ReagentPack™ Subculture Reagents (CC-5034; Lonza), and the cells at passage 7–8 were used for the experiments. HAECs were cultured in Endothelial Cell Basal Medium-2 (EBM-2) (CC-3156; Lonza) containing 0.2% FBS, during adenovirus infection and MG treatment.

### Generation of recombinant adenovirus vectors encoding human GLO1

We generated the recombinant adenovirus expression vector that carries human GLO1 (rAd-GLO1). Human *GLO1* cDNA was obtained from HK2 cells using the following primers: 5′-GGGGTACCATGGCAGAACCGCAGCCC-3′ (forward), 5′-ATAGTTTAGCGGCCGCTACATTAAGGTTGCCATTTTG-3′ (reverse). Recombinant adenovirus vectors were generated using AdEasy™ Adenoviral Vector System (240009; Agilent Technologies, Santa Clara, CA, USA). Briefly, human GLO1 was subcloned into the multicloning site of AdTrack-CMV shuttle vector, which contains green fluorescent protein as reporter gene, in position between Kpn I and Not I. The resultant plasmid was co-transformed into *E. coli* BJ5183 strain cells with the AdEasy-1 adenoviral plasmid. Recombinant bacteria were selected by kanamycin resistance, and recombination was confirmed by PacI endonuclease restriction analysis. Finally the linearized recombinant plasmid was transiently transfected into the packaging 293 cells, using TransIT®-LT1 Transfection Reagent (MIR2304; Mirus Bio LLC, Madison, WI, USA). The culture dishes were incubated at 37 °C for 7–10 days to prepare the primary viral stock. After another two cycles of amplification of recombinant adenovirus vectors in 293 cells, the adenovirus vectors were purified by precipitation with polyethylene glycol followed by two overnights dialysis. The viral titer was determined by limiting dilution assay.

### Transfection

First, HAECs were infected with rAd-GLO1 at a wide range of multiplicity of infection (MOI), from 1 to 50, and measured protein expression and enzymatic activity of GLO1 (Fig. S5A–C). Considering cell viability and levels of expression and activity of GLO1, subsequent experiments were performed at an MOI of 10–20. Approximately 80% confluent HAECs were infected with rAd-GLO1 and harvested 24–48 h postinfection.

### Induction of glycative stress by MG treatment

After 24-h starvation, HAECs were cultured for 24 h in 0.2% FBS in EBM2 containing various concentrations of MG. The cells were harvested for analysis of protein post-translational modification.

### Statistical analysis

After the Smirnov–Grubbs’ outlier test, data were analyzed using graphpad prism version 5.04 for Windows (GraphPad Software, San Diego, CA, USA). All values are expressed as mean ± SEM. Statistical differences between groups were tested using the unpaired Student’s *t*-test, one-way ANOVA, or two- way ANOVA with Bonferroni post hoc tests. A two-sided *P* value of <0.05 was considered statistically significant. For quantitative analyses of immunohistochemistry and Western blotting, values are expressed as relative to the average of mid-age WT rats.

## Abbreviations

CVD: cardiovascular disease
GLO1: glyoxalase I
MG: methylglyoxal
8-OHdG: 8-hydroxydeoxyguanosine
4-HNE: 4-hydroxy-2-nonenal
NO: nitric oxide
eNOS: endothelial nitric oxide synthase
EDR: endothelium-dependent relaxation
AGEs: advanced glycation end-products
CEL: *N*^ε^-(1-carboxyethyl) lysine
CML: carboxymethyllysine
GSH: reduced glutathione
ACh: acetylcholine
SNP: sodium nitroprusside
L-NAME: *N*^G^-nitro-L-arginine methyl ester
PKC: protein kinase C

## Supporting Information

**Fig. S1** PAS staining of aortas from young/mid-age and WT/*GLO1* Tg rats. **Fig. S2** Age-related enhancement in oxidative stress markers, protein carbonyls (A) and 4-HNE (B), were attenuated in thoracic aortas in *GLO1* Tg rats. **Fig. S3** Immunohistochemistry (A) and Western blotting (B) of 3-nitrotyrosine. **Fig. S4** Western blotting of PKC and its phosphorylation. **Fig. S5** Expression and activity of GLO1 in HAECs infected with different MOI of adenovirus vectors.

**Table S1** Lipid metabolism of young/mid-age and WT/*GLO1* Tg rats.
