# Supplementary material for: Glyoxalase I reduces glycative and oxidative stress and prevents age-related endothelial dysfunction through modulation of endothelial nitric oxide synthase phosphorylation
Source: Aging Cell. 2014 Feb 24;13(3):519–28. doi: 10.1111/acel.12204 (PMC4326886; doi:10.1111/acel.12204)

Supplemental figure I.

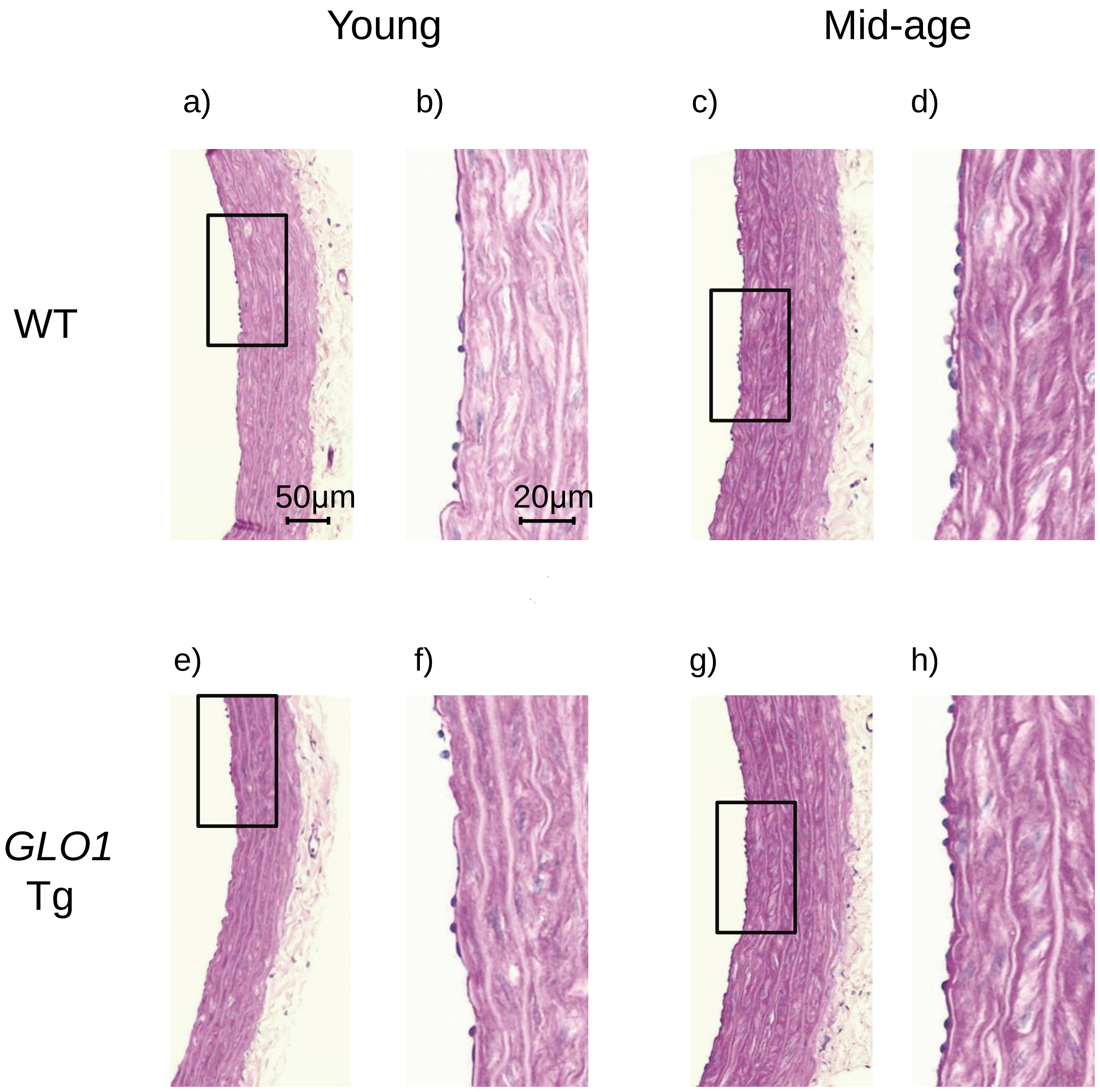

Supplemental figure II.

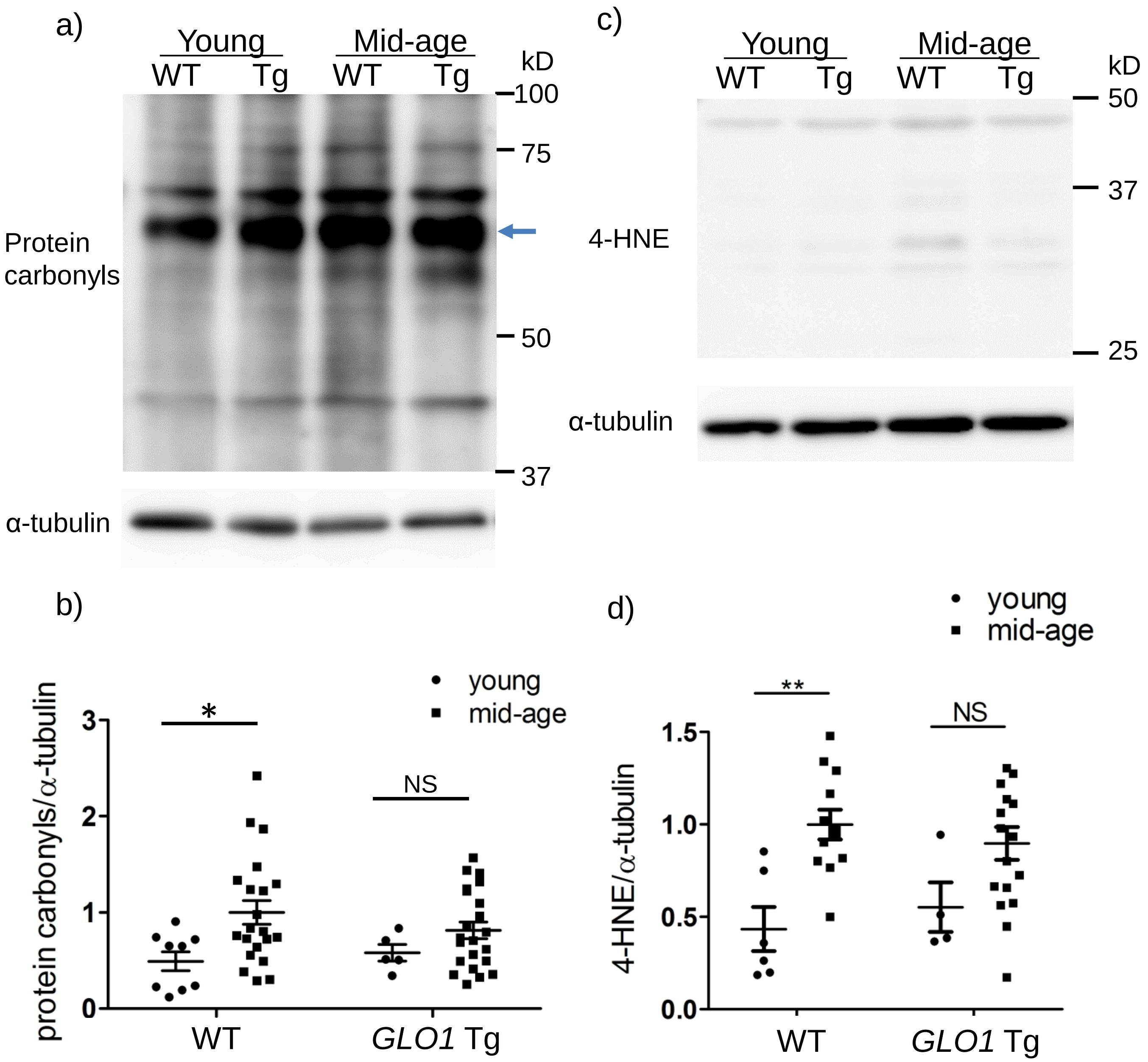

Supplemental figure III.

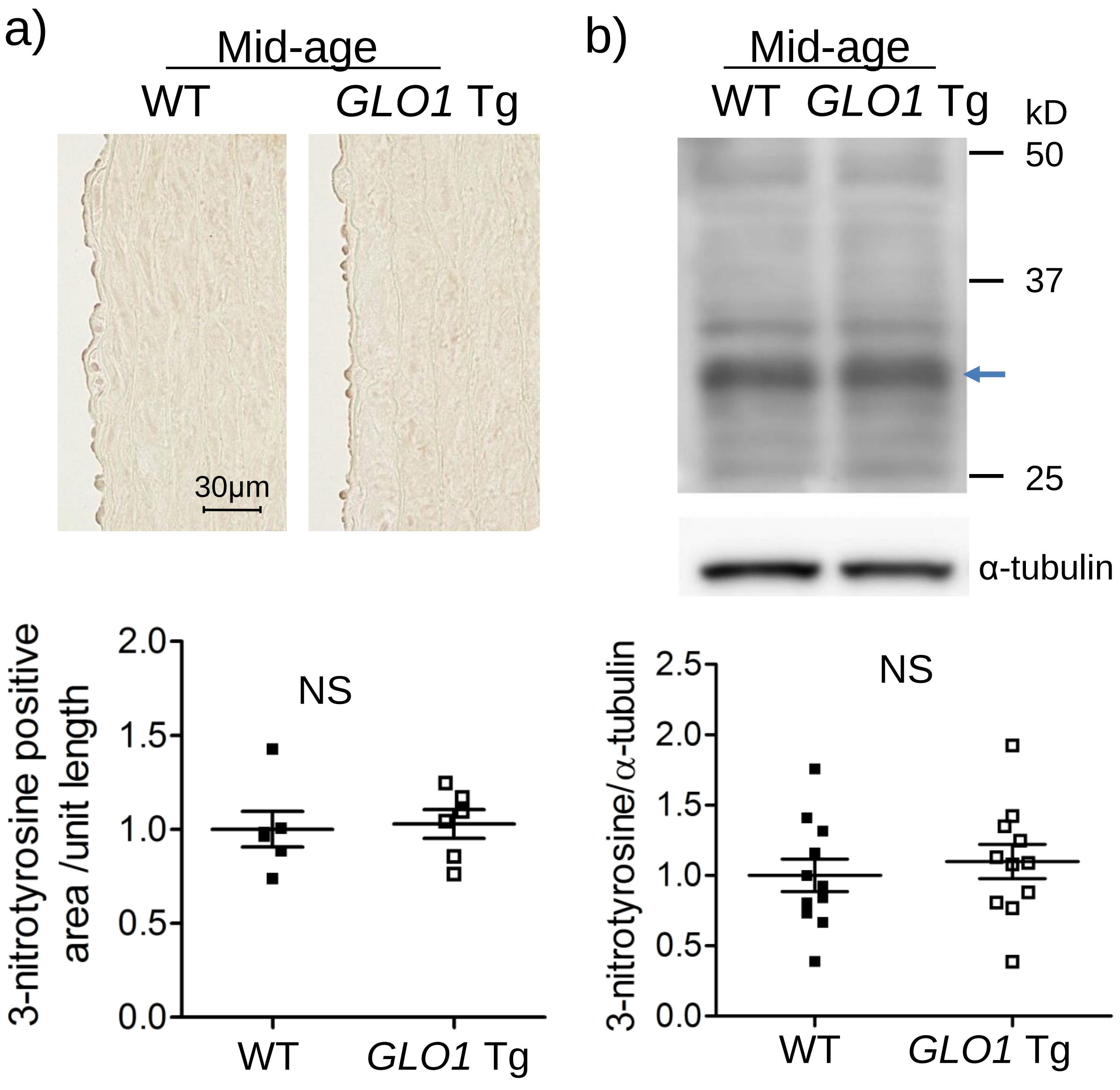

Supplemental figure IV.

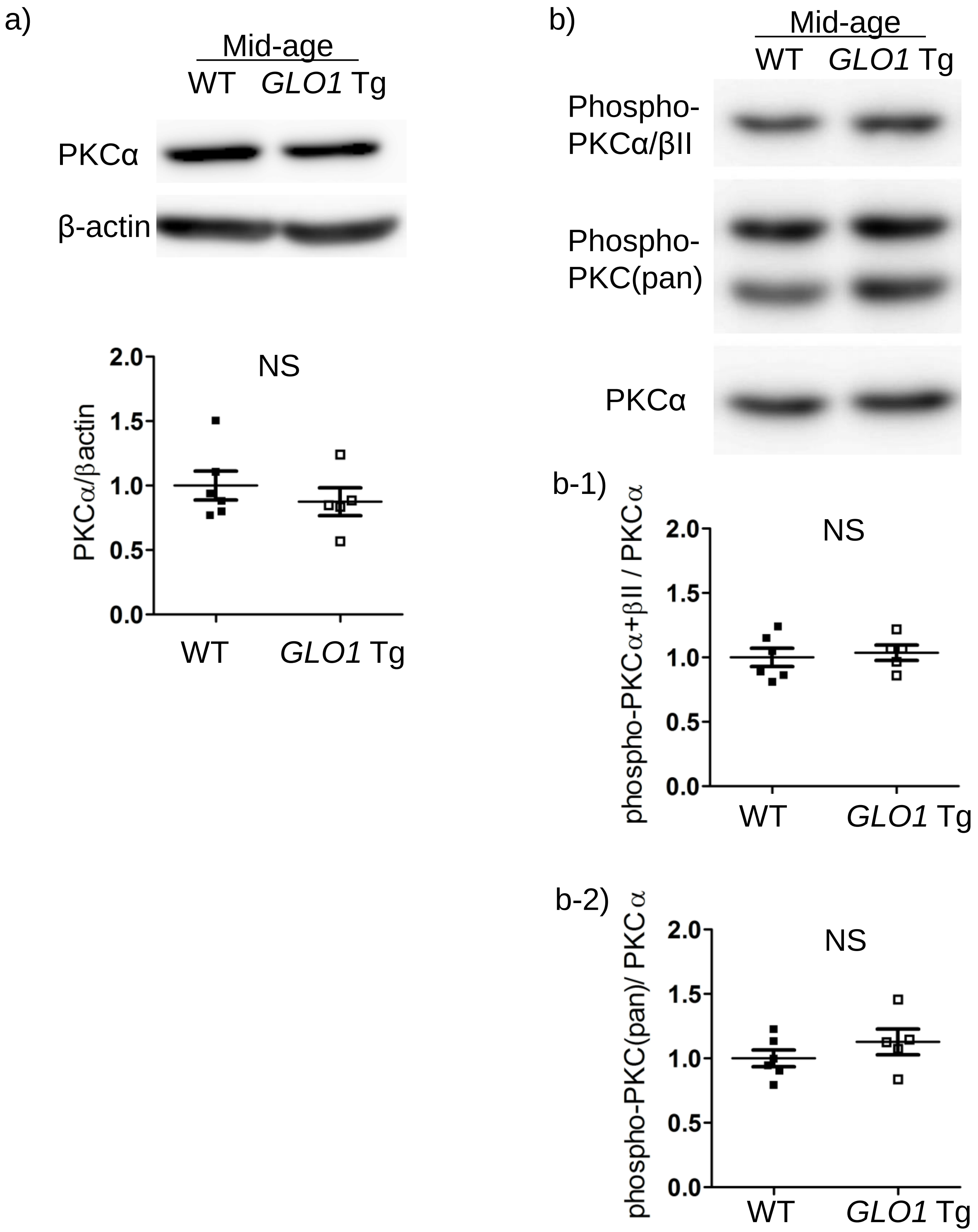

Supplemental figure V.

a)

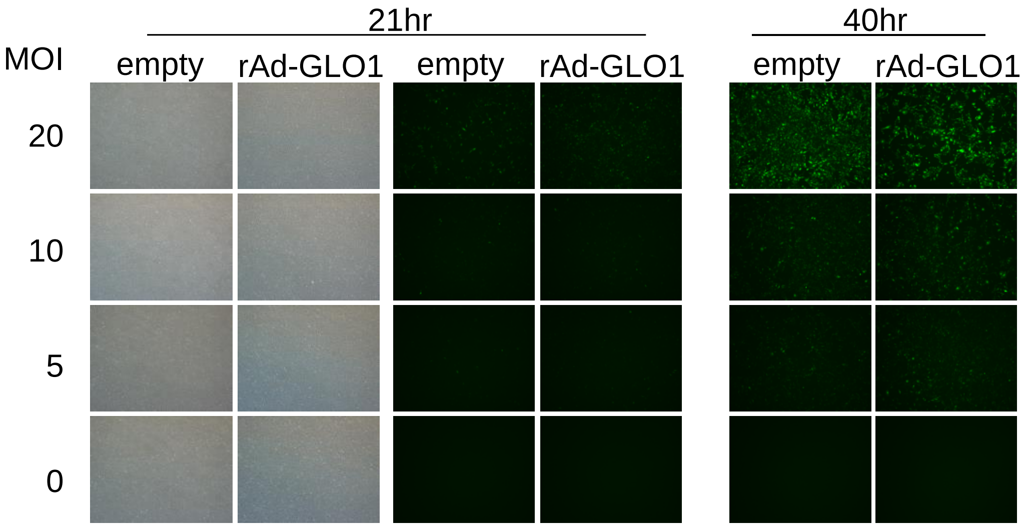

b)

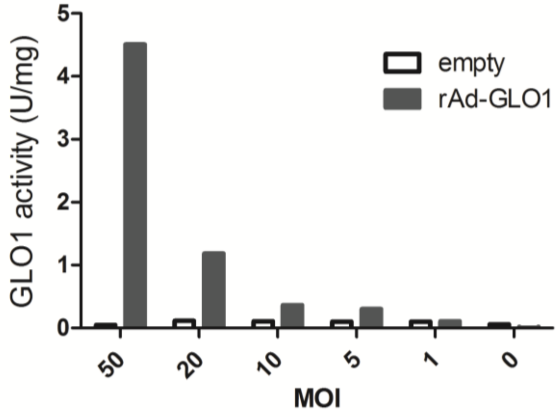

c)

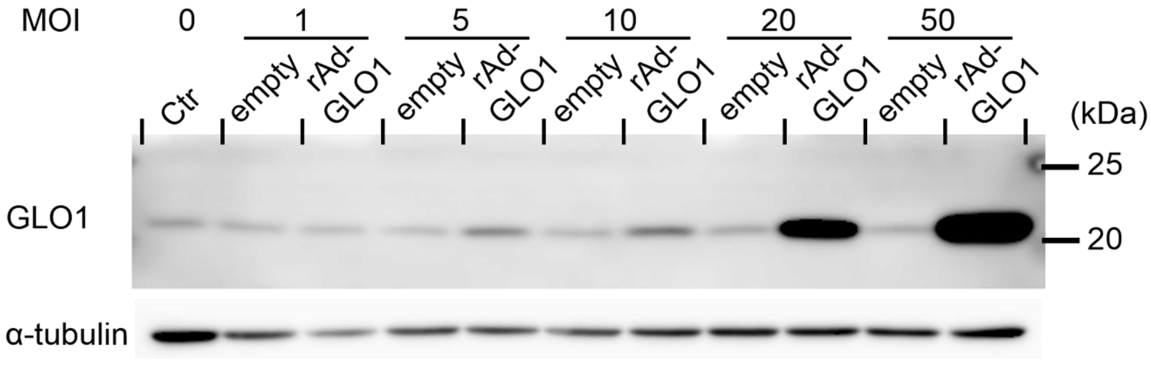

Supplement: Supplementary file 1 — Fig. S1 PAS staining of aortas from young/mid-age and WT/GLO1 Tg rats. Fig. S2 Age-related enhancement in oxidative stress markers, protein carbonyls (A) and 4-HNE (B), were attenuated in thoracic aortas in GLO1 Tg rats. Fig. S3 Immunohistochemistry (A) and Western blotting (B) of 3-nitrotyrosine. Fig. S4 Western blotting of PKC and its phosphorylation. Fig. S5 Expression and activity of GLO1 in HAECs infected with different MOI of adenovirus vectors. [file acel0013-0519-sd1.pdf]
