# Supplementary material for: Glyoxalase I reduces glycative and oxidative stress and prevents age-related endothelial dysfunction through modulation of endothelial nitric oxide synthase phosphorylation
Source: Aging Cell. 2014 Feb 24;13(3):519–28. doi: 10.1111/acel.12204 (PMC4326886; doi:10.1111/acel.12204)
Supplement: Supplementary file 2 — Table S1 Lipid metabolism of young/mid-age and WT/GLO1 Tg rats. [file acel0013-0519-sd2.docx]

Supplemental table I. Lipid metabolism of young/mid-age and WT/*GLO1* Tg rats.

|  | | young | mid-age | age-factor | genotype-  factor |
| --- | --- | --- | --- | --- | --- |
|  |  |  |  |  |  |
| Triglyceride (mg/dl) | WT | 35±9 (4) | 33±11 (10) | ns | ns |
|  | *GLO1* Tg | 29±5 (4) | 24±3 (8) |  |  |
| LDL-cholesterol (mg/dl) | WT | 6±0.4 (4) | 11±1* (11) | *p*<0.001 | ns |
|  | *GLO1* Tg | 9±1 (4) | 12±1 (9) |  |  |
| HDL-cholesterol (mg/dl) | WT | 27±1 (4) | 31±1 (10) | *p*<0.05 | ns |
|  | *GLO1* Tg | 28±1 (4) | 29±1 (8) |  |  |
| Total cholesterol (mg/dl) | WT | 60±4 (4) | 93±7* (10) | *p*<0.001 | ns |
|  | *GLO1* Tg | 66±2 (4) | 90±5 (8) |  |  |

Values are mean ± 1 SEM. Numbers of animals are shown in parentheses.

Two-way ANOVA with post hoc Bonferroni correction was performed. The number of multiple comparison was 2. **p<*0.01, vs. young rats in the same genotype group.
